# Society Meetings

**Published:** 1916-06

**Authors:** 


### SOCIETY MEETINGS

**Brief reports and announcements of meetings of societies of Western New York, and those of general scope, are requested from Secretaries. Copy should be on hand the fifteenth of the month. Full transactions will be published at cost of composition.**

---

**DOCTOR: Is your Society properly represented here? If not, please bring our standing announcement to the attention of the members.**

---

**The Gross Medical Club** held their stated meeting at the Hotel Touraine, Thursday, April 27th, as the guests of Dr. and Mrs. A. G. Bennett, President W. Warren Britt presided.

Dr. A. G. Wilcox, of Tonawanda, read an interesting paper "The Value of the X-ray to the General Practitioner." The essayist spoke from a standpoint of the X-ray in fractures, showing many pictures of unrecognized fractures from clinical evidence, but which were easily and quickly shown by aid of the X-ray plates. He made the unqualified statement that in all cases of injury involving joints or bone, an X-ray picture should be taken as it often saved deformity and trouble for the physician afterward. The paper was discussed quite generally by all present, especially President Britt, who advocated the use of Sodium Silicate bandages instead of plaster of Paris. He said it was easier to get a picture with this method after dressing had been applied, thus it can be shown whether the fracture is in the right position or not. Dr. Britt said the crinoline can be soaked in Sodium Silicate as bought from the drug store, it hardens quite quickly and in every way is as firm as plaster of Paris.

The following cases were reported:

Dr. George F. Cott, man, aged 70, operated upon some time ago for removal of nodes on each side of the neck. Later was seen in consultation on account of slight pain when swallowing. Examination showed an ulcer near the larnyx, about one inch deep, with smooth cartilaginous-like border. The diagnosis of this case he reported as very confusing, having, never seen nor heard of a similar condition, it is either syphilis, tuberculosis or cancer and as different tests are now being made, discussion will be reserved until reports are at hand.

Dr. Baker, Williamsville, a woman, age 45, for three or four years at intervals of three to four months, has suffered severe attacks of pain in the right lower quadrant of the abdomen. She had consulted three doctors who diagnosed appendicitis, who advised operation. At the last attack she consulted the reporter, who took the phosphatic index finding it very low. Compound Phosphorus Mixture (Dowd) was ordered. In three days she was free of pain and has had no recurrence in a year. This was evidently an ovarian neuralgia. He said he could report several cases in which or after which absolute failure in his and other hands by other methods he had used the Phosphatometer and prescribed as to the readings with the most happy results in practically every case.

Dr. McKinney, a case of peri-rectal abscess, operated on six times, always recurred. Dr. McKinney said any condition of this kind meant but one thing, fistula, and the internal opening should be found. The following method, not described in text-books was described, for finding the internal opening of a fistula. If abscess is closed, puncture and let out pus through canula. Wash out cavity thoroughly, then distend with a solution of Methyline blue. With a good light, if the solution does not appear in the rectum and can be seen oozing through the opening, a blue spot will be seen, where the tissue is thin, and is the internal opening.

Dr. J. Henry Dowd, reported a case of priapism of long standing. This is not a disease per se, but a symptom. It is of two varieties, true and false: the true generally accompanied some inflammatory condition of the spinal cord and is very bothersome both day and night. The false, of which the reported case is an example is generally due to some local condition in or around the neck of the bladder or Neuroses. There was no history of venereal disease, attempted examination of the prostate almost threw the patient into a spasm, but it was normal in appearance. Examination of the urine revealed no pathological condition of the urinary tract The phosphatic index registered 90% minus. Fluid extract of valerian, Comp. Phos. Tonic (Dowd) of each two oz. in glycerine for six ounces, a teaspoonful three times a day half an hour after food was ordered.

In ten days, this patient reported great relief, in fact, the priopism had practically disappeared.

This condition might be considered a hyperas thesia of the prostate due to nerve-cell starvation.

Mr. John F. MacHowie entertained the guests during dinner with recitations.

---

**The Alumni Association of the Medical Dept., University of Buffalo,** held its 41st annual meeting, May 30-June 2. This date is too late for a full preliminary announcement and too early for a description in retrospect in this issue. Full transactions will be published in the July or Aug. issue, according to the date received. It is obvious that the Committee in charge have a great many duties to attend to, before, during and for some time after the meeting and that they may not be able to prepare the matter for publication in the July issue.

---

**The Rochester Academy of Medicine** held a regular meeting with Section I, May 10. Dr. Chas. E. Darrow gave a paper on Medical Treatment of Gastric and Duodenal Ulcers; Dr. Edward G. Nugent on Causes and Treatment of Colitis.

---

**The Buffalo Clinical Club** held the last meeting of the season, May 1. Dr. Phillips of Corfu, formerly of the staff of the Ernest Wende Hospital, read a paper on Infectious Diseases; Dr. John H. Pryor read a paper and presented lantern slides of X-ray plates illustrating Immobility of the Diaphragm Following Pleural Effusion. At the last previous meeting, in April, Dr. John M. Lee of Rochester read a paper on radium and exhibited tubes of the element.

---

**The Buffalo Academy of Medicine** has held the following meetings since our previous report: April 26, Section of Pathology, Contraction of the Individual Muscle Fibre, Dr. F. H. Pratt; Immobility of the Diaphragm after Pleural Effusion and Empyema, Dr. John H. Pryor. X-ray pictures by Drs. Leonard Reu and Edward C. Koenig, were shown by lantern.

May 3, Section of Surgery, Rollier Treatment of Bone and Joint Tuberculosis, Drs. Hyde and Lo Grasso of the J. N. Adam Memorial Hospital. Lantern slides and patients were shown.

May 10, Section of Medicine, Acute Abdominal Conditions in Children Demanding Immediate Operation. Dr. A. W. Hengerer.

A joint meeting of the **Binghamton and Elmira Academies of Medicine** was held at the Binghamton State Hospital, May 15. Program:

1. The Reception, Examination and Care of New Admissions, Dr. C. G. Wagner, Superintendent.
2. The Cold Wet Pack, Dr. T. I. Townsend.
3. Brief Resume of What Has Been Done and What is to be Done for the Prevention of Insanity, Drs. Edward Gillespie and R. M. Chapman.
4. Cerebral Arteriosclerosis—Its Clinical and Pathological Aspects—Clinical and Lantern Slide Demonstration, Drs. R. R. Williams and W. J. Tiffany.

Lunch was served at the Hospital.

